# Supplementary material for: Measurement and evaluation of low-carbon tourism development on islands: A case study in Changdao, China
Source: PLoS One. 2025 Jan 13;20(1):e0312490. doi: 10.1371/journal.pone.0312490 (PMC11729940; doi:10.1371/journal.pone.0312490)
Supplement: S1 Data — (DOCX) [file pone.0312490.s001.docx]

**Data1. Data of Island Low Carbon Tourism Development Evaluation Index System**

| **Target** | **Criterions** | **Elements** | **Indicators** | **2016** | **2017** | **2018** | **2019** | **2020** | **2021** |
| --- | --- | --- | --- | --- | --- | --- | --- | --- | --- |
| Comprehensive evaluation index of the level of development of low carbon tourism in the islands ($O)$ | Driving$(C_{1})$ | Economic$(E_{1})$ | Percentage of tertiary industry (%) $I_{1}$ | 35.54 | 36.05 | 36.50 | 36.44 | 31.43 | 31.33 |
|  |  |  | GDP per capita (RMB/person) $I_{2}$ | 155584 | 177885 | 187344 | 193683 | 193883 | 206761 |
|  |  |  | Resident disposable income per capita (RMB) $I_{3}$ | 21455 | 23354 | 25318 | 27502 | 28779 | 31413 |
|  |  |  | Resident consumption expenditure per capita (RMB) $I_{4}$ | 13484 | 14579 | 15731 | 16910 | 17396 | 19819 |
|  |  | Social${(E}_{2})$ | Population size (People) $I_{5}$ | 41990 | 41714 | 41489 | 41286 | 40898 | 41489 |
|  |  |  | Natural population growth rate (‰) $I_{6}$ | 1.16 | -0.36 | 0.02 | 0.14 | -1.73 | -2.48 |
|  |  |  | Urbanization level (%) $I_{7}$ | 53.76 | 53.79 | 53.66 | 53.66 | 53.66 | 53.51 |
|  |  |  | Businesses in the accommodation and catering industry (Unit) $I_{8}$ | 621 | 621 | 609 | 601 | 594 | 179 |
|  |  | Resources$(E_{3})$ | National A-class scenic spots (Unit) $I_{9}$ | 1 | 2 | 2 | 2 | 2 | 2 |
|  |  |  | Forest coverage (%) $I_{10}$ | 51.16 | 50.06 | 49.98 | 49.96 | 46.35 | 60 |
|  |  |  | Coastline length (km) $I_{11}$ | 187.8 | 187.8 | 187.8 | 187.8 | 187.8 | 187.64 |
|  |  |  | CO2 absorption (ton) $I_{12}$ | 89.64 | 90.3 | 80.84 | 84 | 88.96 | 90.86 |
|  |  | Location$(E_{4})$ | Periods of suitable tourist temperatures (day) $I_{13}$ | 144 | 136 | 129 | 130 | 160 | 134 |
|  | Pressure$(C_{2})$ | Market Size$(E_{5})$ | Tourist numbers (10,000 people) $I_{14}$ | 353 | 385 | 381 | 367.7 | 261.55 | 310 |
|  |  |  | Number of people hosted on sea excursions (10,000 people) $I_{15}$ | 15.07 | 16.44 | 17 | 15.6 | 10.84 | 13.24 |
|  |  |  | Electricity consumption (10,000 kWh) $I_{16}$ | 10183 | 10214 | 11069 | 15643 | 16398 | 16266 |
|  |  | Tourism Transportation$(E_{6})$ | Road passenger traffic (10,000 visitors) $I_{17}$ | 112 | 135.41 | 138.73 | 146.53 | 66.57 | 75.9 |
|  |  |  | Marine passenger traffic (10,000 visitors) $I_{18}$ | 224 | 275.15 | 270.82 | 266.97 | 133.13 | 151.8 |
|  |  |  | Road passenger traffic turnover (10,000 visitors km) $I_{19}$ | 3572.8 | 4319.58 | 4425.49 | 4674.31 | 2123.58 | 2421.21 |
|  |  |  | Marine passenger traffic turnover (10,000 visitors km) $I_{20}$ | 6010.00 | 7544.05 | 7112.81 | 7135.67 | 3465.93 | 3805.76 |
|  |  | Tourism Accommodation$(E_{7})$ | Average daily star hotel bed occupancy (Unit) $I_{21}$ | 29 | 32 | 31 | 30 | 21 | 25 |
|  |  |  | Average daily star fisherman’s family home hotel bed occupancy (Unit) $I_{22}$ | 9642 | 10516 | 10407 | 10044 | 7145 | 8468 |
|  |  | Tourism Activities$(E_{8})$ | Number of tourists on sightseeing tours (People) $I_{23}$ | 164.0356 | 178.9057 | 177.0469 | 170.8665 | 121.5397 | 144.0539 |
|  |  |  | Number of visitors on leisure holidays (People) $I_{24}$ | 148.6024 | 162.0735 | 160.3896 | 154.7907 | 110.1047 | 130.5007 |
|  |  |  | Number of visitors on business trips (People) $I_{25}$ | 18.2007 | 19.8506 | 19.6444 | 18.9586 | 13.4855 | 15.9836 |
|  |  |  | Number of visitors for other tourism purposes (People) $I_{26}$ | 23.4004 | 25.5217 | 25.2565 | 24.3748 | 17.3382 | 20.5499 |
|  | State${(C}_{3})$ | Sectoral Carbon Emissions$(E_{9})$ | Total carbon emissions from tourism traffic (10,000 t) $I_{27}$ | 0.6083 | 0.7549 | 0.7303 | 0.745 | 0.3541 | 0.3936 |
|  |  |  | Total carbon emissions from tourism accommodation (10,000 t) $I_{28}$ | 0.6141 | 0.6698 | 0.6627 | 0.6396 | 0.455 | 0.5392 |
|  |  |  | Total carbon emissions from tourism activities (10,000 t) $I_{29}$ | 0.0913 | 0.0995 | 0.0985 | 0.0951 | 0.0676 | 0.0801 |
|  |  | Tourism Industry Carbon Emissions$(E_{10})$ | Total carbon emissions from tourism (10,000 t) $I_{30}$ | 1.3137 | 1.5242 | 1.4915 | 1.4797 | 0.8767 | 1.0129 |
|  |  |  | Carbon emission intensity of tourism (t per10,000 RMB) $I_{31}$ | 0.0339 | 0.0350 | 0.0324 | 0.03102 | 0.0229 | 0.0224 |
|  | Impact$(C_{4})$ | Economic Income$(E_{11})$ | Ticket revenue (Billion RMB) $I_{32}$ | 0.8915 | 0.9500 | 0.8700 | 0.6080 | 0.2849 | 0.3342 |
|  |  |  | Ticket revenue from sea excursions (Billion RMB) $I_{33}$ | 0.3634 | 0.3921 | 0.34 | 0.1921 | 0.1105 | 0.255 |
|  |  |  | Comprehensive tourism revenues (Billion RMB) $I_{34}$ | 38.7 | 43.5 | 46.0 | 47.7 | 38.14 | 45.29 |
|  |  |  | Tourism revenue as a percentage of GDP (%) $I_{35}$ | 61.42 | 61.44 | 62.96 | 64.14 | 51.56 | 57.72 |
|  |  |  | Total retail sales of social consumer goods (10,000 RMB) $I_{36}$ | 133449.9 | 141550.6 | 146997.9 | 155965 | 151457 | 174024 |
|  |  |  | Average annual wage of employees (RMB) $I_{37}$ | 70807 | 75693 | 74596 | 89628 | 91849 | 100580 |
|  |  | Ecological Environment$(E_{12})$ | Air quality conditions - average PM 2.5 concentration (µg/m3) $I_{38}$ | 34 | 34 | 26 | 35 | 32 | 27 |
|  |  |  | Ambient air quality excellent rate (%) $I_{39}$ | 80 | 69 | 75 | 70.4 | 77.8 | 83.3 |
|  |  |  | County ambient air quality composite index $I_{40}$ | 3.8 | 4.1 | 3.5 | 3.3 | 3.6 | 3.38 |
|  |  |  | Sewage treatment rate (%) $I_{41}$ | 97 | 97 | 97 | 97 | 97.4 | 97.4 |
|  |  |  | Green space in built-up areas (acres) $I_{42}$ | 2858.25 | 2918.24 | 2925 | 3195.9 | 3589.8 | 3589.8 |
|  | Response$(C_{5})$ | Government Planning$(E_{13})$ | Energy conservation and environmental protection expenditure (10,000 RMB) $I_{43}$ | 12253 | 12273 | 7297 | 22639 | 25080 | 16779 |
|  |  |  | Number of normative documents related to low carbon development (Unit) $I_{44}$ | 3 | 3 | 4 | 3 | 5 | 5 |
|  |  |  | Establishing low-carbon tourism demonstration areas $I_{45}$ | 3 | 3 | 4 | 5 | 5 | 5 |
|  |  |  | Proportion of low-carbon publicity (%) $I_{46}$ | 3 | 3 | 4 | 4 | 5 | 5 |
|  |  |  | Number of public bus operations (Unit) $I_{47}$ | 40 | 40 | 90 | 90 | 90 | 91 |
|  |  | Corporate Practice$(E_{14})$ | Clean energy usage (%) $I_{48}$ | 3 | 3 | 3 | 4 | 4 | 4 |
|  |  |  | Energy-efficient equipment usage (%) $I_{49}$ | 4 | 4 | 5 | 5 | 5 | 5 |
