# Supplementary material for: Measurement and evaluation of low-carbon tourism development on islands: A case study in Changdao, China
Source: PLoS One. 2025 Jan 13;20(1):e0312490. doi: 10.1371/journal.pone.0312490 (PMC11729940; doi:10.1371/journal.pone.0312490)
Supplement: Attachment — Submitted filename: hhh (1).pdf [file pone.0312490.s003.pdf]

hhh

بواسطة: yuhua Wang

تاريخ الارسال: 14-أغسطس-2024 12:17ص (UTC+0300)
معرف الارسال: 2431671763
اسم الملف: PONE-D-24-10283.docx (76.51K)
حساب الكلمات: 6658
عدد الرموز: 38295

Measurement and evaluation of low-carbon tourism development on islands: A case study in Changdao, China

Mengsha Wang[1, ¶], Jiayu Zuo[2 ¶,*]

1 School of Economy, Shandong Technology and Business University, Yantai, Shandong, China

2 Business School, Hanyang University, Seoul, Korea

* Corresponding author

E-mail address: jyzuo5767@sina.cn

Abstract

China's island tourism is still in the exploratory stage, and the carbon emissions due to island tourism development are still prominent. This study assesses the development of low-carbon tourism of Changdao Island in China. We constructed an evaluation model for low-carbon tourism on islands based on the driver-pressure-state-impact-response model, and the Entropy Weight Method-Analytical Hierarchy Process Method was combined with the weighting method to determine the index weights of every evaluation-indicators. Analysing the annual changes in the development level of low-carbon tourism, the weights of the indicators, and the characteristics of the scores, the low-carbon development mode and key factors of island tourism were explored. The results of the study showed that the indicator "impact" was the most influential element of the island's low-carbon tourism, and the ecological environment value was higher than that of the economic value. Moreover, energy saving, and consumption reduction helped tourists to have a better experience, which further enabled island tourism to have a larger market size. The "response" also occupied a crucial position, where the weighted value of government planning was twice as high as that of corporate practice. The other elements were "pressure", "state", and "driving forces", which showed that low carbon emissions are an important criterion for the island tourism environment and economic factors have the greatest effect in terms of the "driving forces". The study evaluates the level of low-carbon development in island regionals from multiple dimensions, fills the literature gap, and will provide a reference for the study of regional low-carbon and sustainable development of tourism.

1. Introduction

United Nations released the Implementation Schedule for the Ocean Decade", which aims to achieve sustainable development of the ocean by establishing a Common Framework of Global Maritime Interests [1,2]. According to the EU Blue Economic Report 2022 that was issued by the European Commission, the total added value of the blue economy industry reached 184 billion euros in 2022, but rising sea levels may cause huge losses in the future [3]. Moreover, China's "Outline of the 14th Five Year Plan and the Long-term Objectives for 2035" emphasized the revitalization of the rural economy and industries by developing characteristic industries such as rural tourism and homestays, the promotion of a modern marine system, and the development of marine power [4]. Consequently, the development of ocean tourism in coastal areas and islands aligns with the strategic goals of rural revitalization and the development of "maritime power".

During 2012-2021, with the added value of the Marine tourism industry increasing from 33.9% to 44.9%, and it has become a pillar industry for the development of the ocean economy, and it also comprises half of the tourism industry and plays an important role in boosting consumption, domestic demand, and economic growth. Marine tourism has a long industrial chain, which involves the six traditional elements of food, housing, transportation, travel, shopping, and entertainment and the modern development elements of experience, meeting, health, media, organization, and supporting facilities. It also has a strong correlation with upstream and downstream industries and plays a critical role in promoting regional, national, and global economic growth. Moreover, as the offshore fishery resources deplete, island tourism is becoming a powerful engine for rural revitalization, economic growth, and demand expansion in islands.

The rapid development of China's coastal economy has led to a series of marine

environmental problems, such as the increase in marine pollutant emissions and the deterioration of marine ecology in the coastal waters [5]. As an important part of the marine ecosystem, islands are where many plants and animals live, reproduce, and stopover during migration and are ecologically sensitive areas. Therefore, their ecology is more vulnerable to various environmental problems due to human activities. However, although tourism is considered a "green and smoke-free industry", excessive and uncontrolled tourism and development lead to expanded carbon footprints, and the impact of carbon emissions from tourism on the ecological environment and climate change cannot be ignored [6,7,8]. According to the World Tourism Organization Report, tourism has become a "major contributor" to the growth of carbon emissions and its carbon emissions continue to grow.

The investigation into energy consumption and carbon emissions within the tourism sector has been a prominent subject of interest among scholars in recent years. As early as the 1990s, the issue of energy consumption in the tourism industry, as exemplified by the Hawaiian Islands [9], began to be studied. Since then, top-down method [10,11,12], bottom-up method [13,14,15], TSA [16,17], LCA [18] and other methods have been used to conduct the assessment of tourism carbon emissions. In addition, the LMDI method has been used to analyse the impact factors of tourism carbon emissions from the perspective of the life cycle of tourist destinations, arguing that the tourist scale effect and energy intensity contribute to the positive growth of carbon emissions [19]. Furthermore, the carbon intensity factor, the income structure factor, the consumption level factor, and the tourism scale factor have been considered to be the four factors affecting the tourism carbon emissions [20]. Most of the literature focused on the estimating carbon emissions but few prior studies have focused on the measurement of the overall green and low-carbon development

level of island tourism and providing possible measures to enhance zero-carbon development, we attempted to explore these aspects.

In the context of the demand for low-carbon tourism development and the constraint of the ecological carrying capacity of islands, this study created a low-carbon island tourism development level evaluation index system to measure and evaluate the development level of low-carbon island tourism and explore how different elements such as the economy, society, the environment, resources, and industry, in the island tourism system are integrated and developed. This study aimed to provide a theoretical basis for the green, low-carbon, and sustainable development goals of island tourism, contribute to the healthy economic and social development of islands, and promote low-carbon tourism for the revitalization of fishing villages on islands. The study could be used as a basis for contributing toward achieving sustainable development in the global ocean economy and the development and construction of international zero-carbon islands in China. Moreover, island tourism destinations are relatively independent geographical units and special tourism destinations, and thus exploring their low-carbon development modes for scientific policy control, construction planning and the construction of "zero-carbon" demonstration zones can provide a basis for the construction of zero-carbon communities, low-carbon towns, and low-carbon cities.

2. Literature Review

Most of the empirical studies on the evaluation of island tourism development in China have analysed the coupling coordinated development level of the island tourism systems by constructing an evaluation index system for all or two of the three factors, namely, tourism, the

socio-economy, and the ecological environment. The results of these studies showed that the level of coupling and coordination between island tourism and the ecological environment [21,22] economic growth [23], and socio-economics [24] in China is not high but is gradually increasing. The tourism system is a complex integrated dynamic system that is composed of multiple elements, and its green and low-carbon development requires the organic integration and coordination of various aspects, such as the economy, society, and environment. Constructing a low-carbon tourism development evaluation index system is an important step in the transition from theoretical research to practical application, and it is an effective way to achieve economic, social, and ecological environmental benefits. It is also a key part of relevant policy formulation and construction planning management [25,26,27].

Studies on the low-carbon aspects using the DPSIR model have focused on low-carbon cities and transportation. By integrating the advantages of the pressure-state-response (PSR) model and the driving force-state-response (DSR) model and considering the five major elements (economy, society, resources, environment, and policy), the driver-pressure-state-impact-response (DPSIR) model is more comprehensive, systematic, adaptive, and holistic, and it is widely used in sustainability and low carbon research(Shao et al. [25]; Liu [28]; Zhang et al. [29]). Additionally, a low-carbon green transportation evaluation index system was established and employed for the analysis of the urban green and low-carbon transportation development level and its main influencing factors in Dalian [30], Xuzhou [31], Beijing [32], and Xi'an [33]. This provided a theoretical basis for policy formulation. Then studies on island systems using the DPSIR model have mainly focused on the two aspects of ecological safety (Li et al. [34]) and sustainable development (Tian et al. [35]). However, no research has been conducted on low-carbon island

tourism development by constructing a low-carbon tourism development level evaluation index system based on the DPSIR model with island systems as the focus and carbon emissions as a specific index.

Consequently, this study conducted a comprehensive analysis of ~~the causal~~ links between the economy, society, resources, environment, policies, and carbon emissions that were related to island tourism development from a system analysis perspective. Based on the DPSIR analysis model and with carbon emissions from tourism as the core index, this study constructed an index system that reflects the overall development level of low-carbon island tourism. The index included five sub-systems of the low-carbon tourism system, that is, its potential driving forces, direct explicit pressure, core carbon source department state, socio-economic environment impact, and government and enterprise response. The low-carbon tourism development level of Changdao Island was analysed, which could provide theoretical guidance and support for the construction of a comprehensive marine ecological civilization pilot zone and the formulation of low-carbon development policies for Changdao Island.

3. Methods

3.1 Research Design

First, we divided the major tourism departments and calculated the carbon emissions of each department separately. Secondly, in order to comprehensively assess the development level of tourism carbon emissions in Changdao Island, we set five criterion layers, namely driving, pressure, state, impact and response, according to the DPSIR model. Within these criterion layers, they correspond to various kinds of small indicators. Then, the basic data of various indicators in

the system from 2016 to 2021 were collected, and their characters were judged according to their positive or negative impacts on tourism carbon emissions. Thirdly, according to characters and data of each year, we obtained the information entropy and the weights of these indicators in the total system by the entropy weighting method. Meanwhile, in order to ensure the comprehensiveness of the system, we also obtained the AHP weights through the AHP method based on the ratings of several experts on different carbon emission indicators. Fourth, the entropy weights and AHP weights are combined in equal proportions to obtain the comprehensive weights of the overall carbon emission environmental system. Fifth, the entropy weights change according to the statistical data of each year, and the AHP weights of the expert scores are consistent every year. After synthesizing the two, the weights of each indicator are summarized every year, and finally the scoring value of each criterion layer of the environment system was obtained for the carbon emission impact indicators of the system. These score values are yearly changing comprehensive assessments of the whole low-carbon island tourism emission system.

3.1.1 Tourism Carbon Emissions Measurement

Previous studies on carbon emissions from different tourism departments revealed that tourism transportation, tourist accommodation, and tourism activities accounted for more than 90% of carbon emissions from tourism, indicating that they showed a long tail effect. Hence, the carbon emissions from tourism were measured according to the principle of "Occam's razor", with the carbon emissions of the three core carbon source departments as the measurement objects [24]. Consequently, the carbon emissions from tourism were calculated as follows:

$$C = \sum_{i=1}^{n} C_i \tag{1}$$

$$C_t = \sum_{i=1}^{n} p_{ti} d_i k_{ti} \quad (2)$$

$$C_h = 365 \sum_{i=1}^{n} q_i r_i \beta_i e \quad (3)$$

$$C_a = \sum_{i=1}^{n} p_{ai} k_{ai} \quad (4)$$

where C is the total carbon emissions from tourism, C_i is the total carbon emissions from the different tourism departments, C_t is the total carbon emissions from tourism transportation, C_h is the total carbon emissions from tourist accommodations, and C_a is the total carbon emissions from tourism activities; q_i is the number of beds in the accommodation mode i, r_i is the average daily occupancy rate of the beds in the accommodation mode i, and β_i is the unit energy consumption of the accommodation mode i (in MJ/night); e is the conversion coefficient of the energy consumption and carbon emissions (in g/MJ); p_{ai} is the number of tourists choosing the tourism activity i, and k_{ai} is the carbon emissions from the tourism activity i (in g/capita).

In terms of the statistics of the carbon emissions from tourism transportation, to accurately estimate the carbon emissions from tourism transportation for off-shore island tourism destinations, the "central transportation and internal transportation to the scenic spot" was specified by the narrow statistical boundary and was used as the statistical object [36]. Based on the waterway tourist turnover, road tourist turnover, and field research, the proportion of tourists that chose each transportation mode was obtained. Based on the obtained data, the tourist turnover ($p_{ti}d_i$ in Eq (2)) of each transportation mode was calculated. Then, according to the k_{ti} carbon emission coefficient, the carbon emissions of each transportation mode were obtained. In terms of carbon emissions from tourism accommodation, considering that some of the fishtainment homestays were ultra-small-scale businesses that were self-owned by families and considering the

availability of the statistical data, the measured carbon emissions from tourist accommodation in Changdao Island were limited to star-rated fishtainment homestays and hotels. The carbon emissions from tourist accommodation were calculated using Eq. (3). In terms of the carbon emissions from tourism activities, according to the analysis of the tourist motivation based on the accommodation reviews, the proportion of tourists for each type of tourism activity and the corresponding person-times were determined.

3.1.2 Low Carbon Tourism Evaluation Index System Construction

Driver-pressure-state-impact-response Model. When compared with the PSR model and the DSP model, the DPSIR model comprehensively covers and integrates the five elements of economy, society, resource, environment, and policy. It can also give a clear, complete, and dynamic reflection of the causal, constraint, and feedback relationships of the five indexes, namely driving forces, pressure, state, impact, and response, by establishing a complete D-P-S-I-R causality chain from the perspective of system analysis. This model was initially used in sociological research, but it has gradually been used in the fields of sustainable development and ecological environment, and now it has been applied to studies to evaluate the development of cities, transportation, and other research objects in the low-carbon field.

Construction of an Evaluation System. To investigate the relationship between tourism, socio-economic development, and carbon emissions in tourism-based island systems and measure the island tourism low-carbon development level, an evaluation index system was constructed that included an objective layer for the overall evaluation of the island tourism low-carbon

development level, five criterion layers, namely, driving forces, pressure, state, impact, and response, the element layers of all the criterion layers, and an indicators layer for specific measurement (Fig 1). The driving forces drive the development of island tourism and are the potential cause for an increase or reduction in carbon emissions. Based on existing studies, four driving elements (economy, society, resource, and location) and 21 indexes were preliminarily determined. The pressure is an explicit index that is directly affected by the development of island tourism under the internal action of the driving forces, and it has an impact on the carbon emissions of the islands. According to the analysis of carbon emissions from tourism, four element layers (island tourism market size, island transportation, island accommodation, and island tourism activity) and 16 indexes were preliminarily determined. The state is the carbon emission state of island tourism and its sub-sectors of transportation, accommodation, and tourism activity under the internal driving forces and direct pressures, and two element layers (tourism and sub-sectors) and five indexes were preliminarily determined. The impact is the impact on the economic income and ecological environment of the islands due to carbon emissions from island tourism, and two element layers and 14 indexes were preliminarily determined. The response is the response to the economic, social, and ecological impacts on the islands, mainly in terms of the measures that were taken to promote low-carbon island tourism development, and two element layers (government [macro-control side] and enterprises [intermediary side]) and 10 indexes were preliminarily determined. There were 14 element layers and 86 indexes in total. After the elimination of inappropriate, duplicate, and controversial indexes through expert consultation and statistical analysis and those with missing data based on data query and consultation, a total of 14 element layers and 49 indexes were finally determined in table 1.

Fig 1. Driver-pressure-state-impact-response model for the evaluation of low-carbon tourism development in islands

Table 1 Island Low Carbon Tourism Development Evaluation Index System

Target	Criterions	Elements	Indicators	Character
Comprehensive evaluation index of the level of development of low carbon tourism in the islands (O)	Driving(C_1)	Economic(E_1)	Percentage of tertiary industry (%) I_1	Positive
			GDP per capita (RMB/person) I_2	Positive
			Resident disposable income per capita (RMB) I_3	Positive
			Resident consumption expenditure per capita (RMB) I_4	Positive
		Social(E_2)	Population size (People) I_5	Positive
			Natural population growth rate (‰) I_6	Positive
			Urbanization level (%) I_7	Positive
			Businesses in the accommodation and catering industry (Unit) I_8	Positive
		Resources(E_3)	National A-class scenic spots (Unit) I_9	Positive
			Forest coverage (%) I_{10}	Positive
			Coastline length (km) I_{11}	Positive
			CO2 absorption (ton) I_{12}	Positive
		Location(E_4)	Periods of suitable tourist temperatures (day) I_{13}	Positive
	Pressure(C_2)	Market Size(E_5)	Tourist numbers (10,000 people) I_{14}	Positive
			Number of people hosted on sea excursions (10,000 people) I_{15}	Positive
			Electricity consumption (10,000 kWh) I_{16}	Inverse

	Tourism Transportation(E_6)	Road passenger traffic (10,000 visitors) I_{17}	Positive
		Marine passenger traffic (10,000 visitors) I_{18}	Positive
		Road passenger traffic turnover (10,000 visitors km) I_{19}	Positive
		Marine passenger traffic turnover (10,000 visitors km) I_{20}	Positive
	Tourism Accommodation (E_7)	Average daily star hotel bed occupancy (Unit) I_{21}	Positive
		Average daily star fisherman's family home hotel bed occupancy (Unit) I_{22}	Positive
	Tourism Activities(E_8)	Number of tourists on sightseeing tours (People) I_{23}	Positive
		Number of visitors on leisure holidays (People) I_{24}	Positive
		Number of visitors on business trips (People) I_{25}	Positive
		Number of visitors for other tourism purposes (People) I_{26}	Positive
State(C_3)	Sectoral Carbon Emissions(E_9)	Total carbon emissions from tourism traffic (10,000 t) I_{27}	Inverse
		Total carbon emissions from tourism accommodation (10,000 t) I_{28}	Inverse
		Total carbon emissions from tourism activities (10,000 t) I_{29}	Inverse
	Tourism Industry Carbon Emissions(E_{10})	Total carbon emissions from tourism (10,000 t) I_{30}	Inverse
		Carbon emission intensity of tourism (t per10,000 RMB) I_{31}	Inverse

		Ticket revenue (Billion RMB) I_{32}	Positive
		Ticket revenue from sea excursions (Billion RMB) I_{33}	Positive
		Comprehensive tourism revenues (Billion RMB) I_{34}	Positive
	Economic Income(E_{11})	Tourism revenue as a percentage of GDP (%) I_{35}	Positive
		Total retail sales of social consumer goods (10,000 RMB) I_{36}	Positive
Impact(C_4)		Average annual wage of employees (RMB) I_{37}	Positive
		Air quality conditions - average PM 2.5 concentration (μg/m3) I_{38}	Inverse
		Ambient air quality excellent rate (%) I_{39}	Positive
	Ecological Environment(E_{12})	County ambient air quality composite index I_{40}	Positive
		Sewage treatment rate (%) I_{41}	Positive
		Green space in built-up areas (acres) I_{42}	Positive
		Energy conservation and environmental protection expenditure (10,000 RMB) I_{43}	Positive
		Number of normative documents related to low carbon development (Unit) I_{44}	Positive
Response(C_5)	Government Planning(E_{13})	Establishing low-carbon tourism demonstration areas I_{45}	Positive
		Proportion of low-carbon publicity (%) I_{46}	Positive
		Number of public bus operations (Unit) I_{47}	Positive
		Clean energy usage (%) I_{48}	Positive

Corporate Practice(E_{14})	Energy-efficient equipment usage (%) I_{49}	Positive

Abbreviations: GDP, Gross Domestic Product.

Determination of Index Weights. After the data from the low-carbon island tourism development evaluation index system were obtained, the raw data of the varying attributes and dimensions were nondimensionalized to obtain standardized and comparable data. The extreme value processing method was used to substitute the positive and negative indexes into Eq. (5) and Eq. (6) as follows:

$$X_{ij} = \frac{I_{ij} - I_{min}}{I_{max} - I_{min}} \quad (5)$$

$$X_{ij} = \frac{I_{max} - I_{ij}}{I_{max} - I_{min}} \quad (6)$$

Since the Analytical Hierarchy Process (AHP) method requires experts to compare the importance of one index with another index for each layer and then assign weights subjectively according to the scale table, it has the disadvantage of being influenced by subjective factors. However, the entropy weight method also has the disadvantage of purely objective weighting. Hence, this study used the Entropy Weight Method-AHP combined with the weighting method to determine the index weights, which reduced the disadvantages of both methods and had the advantages of high rationality, systematicness, and accuracy.

A judgment matrix was constructed for the criterion layers and five indicator layers based on the export scores. As seen in Eqs. (7) - (10), the AHP weights of the criterion layers and indicator layers were obtained. Also, a consistency test was performed on the judgment matrix.

$$w_i^0 = \frac{(\prod_{j=1}^{n} a_{ij})^{\frac{1}{n}}}{\sum_{i=1}^{n} (\prod_{j=1}^{n} a_{i,j})^{\frac{1}{n}}} \quad (i = 1,2,3 \ldots, n) \quad (7)$$

$$\lambda_{max} = \frac{1}{n}\sum_{i=1}^{n}\frac{A\,\rho}{w_i} \quad (8)$$

$$CI = \frac{\lambda_{max}-n}{n-1} \quad (9)$$

$$CR = \frac{CI}{RI} \quad (10)$$

where w_i^0 is the relative weight of the element, A is the judgment matrix, a_{ij} is the weight vector, and n is the number of indexes; λ_{max} is the maximum eigenvalue, CI is the consistency index, RI is the average random consistency index, and CR is the consistency ratio.

Based on the standardized data that were obtained by the nondimensionalization of the raw objective data, the entropy weight method was used to conduct a comprehensive evaluation of multiple indexes and calculate the index variability, information entropy, information entropy redundancy, index weights, and comprehensive score using Eqs. (11) - (14), and finally the weight of each index was obtained in Table 2.

$$P_{ij} = \frac{X_{ij}}{\sum_{i=1}^{n} X_{ij}} \quad (i = 1,2,3 \ldots, n; j = 1,2,3 \ldots, m) \quad (11)$$

$$e_j = -\frac{1}{\ln n}\sum_{i=1}^{n} P_{ij}\ln P_{ij} \quad (12)$$

$$g_j = 1 - e_j \quad (13)$$

$$w_j = \frac{g_j}{\sum_{j=1}^{m} g_j} \quad (14)$$

where P_{ij} is the weight of i to j, e_j is the information entropy, g_j is the information entropy redundancy, and w_j is the index weight obtained by the entropy weight method; n is the number of indexes and m is the number of years.

Table 2 Indicator Weights

Target	Criterions	Elements	Indicators	Entropy	AHP	Combined
(Weight)	(Weight)	(Weight)		method weight	method weight	weight
O(1)	C_1(0.1469)	E_1(0.0450)	I_1	0. 0209	0. 0054	0. 0131

		I_2	0.0117	0.0043	0.0080
		I_3	0.0165	0.0069	0.0117
		I_4	0.0173	0.0069	0.0121
	$E_2(0.0415)$	I_5	0.0130	0.0072	0.0101
		I_6	0.0149	0.0072	0.0110
		I_7	0.0123	0.0090	0.0107
		I_8	0.0099	0.0094	0.0096
	$E_3(0.0420)$	I_9	0.0099	0.0053	0.0076
		I_{10}	0.0196	0.0108	0.0152
		I_{11}	0.0099	0.0046	0.0073
		I_{12}	0.0130	0.0108	0.0119
	$E_4(0.0184)$	I_{13}	0.0312	0.0057	0.0185
$C_2(0.1917)$	$E_5(0.0517)$	I_{14}	0.0122	0.0156	0.0139
		I_{15}	0.0122	0.0156	0.0139
		I_{16}	0.0292	0.0184	0.0238
	$E_6(0.0626)$	I_{17}	0.0176	0.0136	0.0156
		I_{18}	0.0171	0.0136	0.0153
		I_{19}	0.0176	0.0136	0.0156
		I_{20}	0.0185	0.0136	0.0160
	$E_7(0.0259)$	I_{21}	0.0124	0.0136	0.0130
		I_{22}	0.0122	0.0136	0.0129
	$E_8(0.0515)$	I_{23}	0.0122	0.0135	0.0129

		I_{24}	0.0122	0.0135	0.0129
		I_{25}	0.0122	0.0135	0.0129
		I_{26}	0.0122	0.0135	0.0129
C_3(0.1699)	E_9(0.1037)	I_{27}	0.0342	0.0371	0.0356
		I_{28}	0.0309	0.0371	0.0340
		I_{29}	0.0312	0.0371	0.0341
	E_{10}(0.0662)	I_{30}	0.0321	0.0371	0.0346
		I_{31}	0.0261	0.0371	0.0316
C_4(0.2828)	E_{11}(0.1321)	I_{32}	0.0196	0.0269	0.0233
		I_{33}	0.0141	0.0269	0.0205
		I_{34}	0.0195	0.0269	0.0232
		I_{35}	0.0112	0.0269	0.0191
		I_{36}	0.0173	0.0269	0.0221
		I_{37}	0.0222	0.0254	0.0238
	E_{12}(0.1507)	I_{38}	0.0275	0.0290	0.0282
		I_{39}	0.0190	0.0289	0.0240
		I_{40}	0.0223	0.0290	0.0256
		I_{41}	0.0592	0.0290	0.0441
		I_{42}	0.0286	0.0290	0.0288
C_5(0.2088)	E_{13}(0.1448)	I_{43}	0.0168	0.0321	0.0245
		I_{44}	0.0398	0.0319	0.0358
		I_{45}	0.0238	0.0321	0.0279

	I_{46}	0. 0250	0. 0319	0. 0284
	I_{47}	0. 0220	0. 0345	0. 0282
E_{14}(0.0640)	I_{48}	0. 0375	0. 0343	0. 0359
	I_{49}	0. 0220	0. 0343	0. 0281

Additionally, by substituting the AHP weights and the entropy weight method weights into Eq (15), the comprehensive weight w of the indexes was obtained (Table 2). Using Eq (16), the final score of the low-carbon island tourism development level evaluation was calculated.

$$w = 0.5w_i + 0.5w_j \quad (15)$$

$$S_i = \sum_{j=1}^{m} w X_{ij} \quad (16)$$

3.2 Data Source

The raw data that were used in this study to measure the carbon emissions from tourism and to construct a low-carbon tourism development evaluation system were obtained from the Zhong Guo Hai Dao Zhi, Statistical Yearbook of Yantai, Statistical Yearbook of Changdao Island, Changhai County Statistics Bulletin of National Economic and Social Development, Changdao Island Comprehensive Experimental Zone Statistics Bulletin of National Economic and Social Development, and other government websites. The government departments of the Changdao Island Comprehensive Experimental Zone Office, such as the Working Committee and Management Committee, the Transportation and Housing Construction Bureau, the Ministry of Publicity, the Ministry of Culture and Tourism, and the Bureau of Ecology and Environment, provided data support. The accommodation data, such as hotel reviews from the Ctrip website,

were collected using the Bazhuayu web crawler. Additionally, some of the data were based on the results of previous studies at home and abroad or obtained from field research.

4. Results

4.1 Carbon Emissions from Tourism

According to table 3, from 2016 to 2021, the total carbon emissions from tourism in Changdao Island showed a decreasing trend, which was related to the clean energy input and low-carbon transformation but there was also a close correlation with the downsizing of tourism. From 2016 to 2021, the annual carbon emissions from tourism were 1.3135, 1.5244, 1.4915, 1.4796, 0.8766, and 1.013 Mt, respectively.

Table 3 Annual Tourism Carbon Emissions by Sector

Unit: Mt

Item \ Year	2016	2017	2018	2019	2020	2021
**Transportation**	0.6083	0.7549	0.7303	0.7450	0.3541	0.3936
Ship and ferry	0.4207	0.5280	0.4978	0.4995	0.2426	0.2664
Fuel car	0.1769	0.2138	0.2191	0.2314	0.1051	0.1198
New energy car	0.0017	0.0022	0.0022	0.0023	0.0011	0.0012
Bus	0.0090	0.0109	0.0112	0.0118	0.0054	0.0061
Cycling and walking	0	0	0	0	0	0
**Accommodation**	0.6140	0.6699	0.6628	0.6396	0.4549	0.5392

Star-rated fisherman's family home hotel	0.6081	0.6633	0.6564	0.6335	0.4506	0.5341
Star-rated hotel bed occupancy	0.0059	0.0066	0.0064	0.0061	0.0043	0.0051
**Activities**	0.0912	0.0996	0.0984	0.0950	0.0676	0.0802
Sightseeing tour	0.0186	0.0203	0.0201	0.0194	0.0138	0.0164
Leisure holiday	0.0676	0.0738	0.0730	0.0704	0.0501	0.0594
Business trip	0.0039	0.0043	0.0042	0.0041	0.0029	0.0034
Other tourism purposes	0.0011	0.0012	0.0012	0.0011	0.0008	0.0010
Total	1.3135	1.5244	1.4915	1.4796	0.8766	1.013

Changdao Island is an off-shore island tourism destination and the central transportation mode that is used to reach the scenic spot is waterway transportation. Since 2018, to promote the construction of marine ecological civilization and to realize green travel on Changdao Island, there has been a ban on non-local vehicles from entering the island. Hence, the internal road transportation modes that were available for tourists on the island included leased cars, operating fuel cars, electric cars, buses, sightseeing vehicles, bicycles, and walking. In this study, the carbon emissions of the available internal transportation modes were as follows [35]: 0.07 kg/pkm for ships and ferries, 0.075 kg/pkm for fuel cars, 0.01 kg/pkm for electric cars, 0.018 kg/pkm for buses/sightseeing vehicles, and 0 kg/pkm for bicycles/walking. As the only way for tourists to enter the island, water transport was the main source of carbon emissions from tourism transportation, accounting for 67% -70%. During 2016-2019, with the change in the number of tourists, the tourist turnover for each transportation mode fluctuated slightly, and the carbon emissions from tourism transportation also showed a relatively stable trend. During the COVID-

19 outbreak, tourism was drastically affected. Specifically, the number of tourists dropped significantly, the tourist turnover was reduced by about 50%, and the total carbon emissions from tourism transportation were reduced to 0.3541 Mt (2020) and 0.3936 Mt (2021) shown in table 3.

In 1999, the "fishtainment" tourism model emerged in Changdao Island and Penglai, with fishermen's families in fishing villages as the main operation entity, and it integrated the experience of fishermen's folk customs, accommodation, catering, and leisure, with tourism and featured fishermen's lifestyle and fishing culture. Herein, the unit energy consumption of a star-rated fishtainment homestay was 40 MJ/bed-night [36], the unit energy consumption of star-rated hotels was 130 MJ/bed-night [22], and the conversion coefficient between energy consumption and carbon emissions was set to 43.2 g/MJ [7,37]. With the transition of tourists' tourism purpose to leisure and vacation, the accommodation and catering features of fishtainment become important, and fishtainment homestays became the major choice for overnight tourists in Changdao Island. Thus, they are also the main source of carbon emissions from tourist accommodation. During 2016-2018, with the increase in the number of overnight tourists, the carbon emissions increased; during 2018-2020, the carbon emissions from tourist accommodation continuously decreased; during 2020-2021, the carbon emissions from tourist accommodation increased significantly.

Based on previous studies [33,38] and the main tourism purpose of the Changdao Island tourists, the tourism activities and their unit energy consumption and carbon emissions were determined as follows: sightseeing (energy consumption: 8.5 MJ/capita; carbon emissions: 113.6 g/capita), leisure vacation (energy consumption: 26.58.5 MJ/capita; carbon emissions: 455.0 g/capita), business trip (energy consumption: 16 MJ/capita; carbon emissions: 214.2 g/capita), and

other (energy consumption: 3.5 MJ/capita; carbon emissions: 46.9 g/capita). On this basis, the carbon emissions from the tourism activities of Changdao Island tourists during 2016-2021 were obtained in table 3. The total carbon emissions from tourism activities were 0.0912, 0.0996, 0.0984, 0.0950, 0.0676, and 0.0802 Mt, respectively, during 2016-2021.

4.2 Results of the Evaluation of the Low-Carbon Tourism Development in the Islands

In this section, according to the established low-carbon island tourism development level evaluation index system, the weight of each layer was obtained in table 2, and the annual comprehensive score was obtained in fig 2. Additionally, the annual variation in the low-carbon tourism development level, index weights, and score features were analyzed to explore the development pattern and key factors of low-carbon tourism development.

SFig 2. Results of the evaluation of low carbon tourism in Changdao Island.

The "impact" was the most influential element of the island low-carbon tourism valuation with a weighted value of 0.2828. The "impact" indicator expressed the feedback role of the changes in the state of the island due to low-carbon tourism development on the ecological environment and economy. At the "impact" level, the ecological environment value was 0.1507 higher than that of the economic value (0.1321). This explains that the development of low-carbon tourism promoted regional air quality, sewage treatment, and green spaces. Apart from the environmental state, economic incomes, such as local employee earnings, ticket revenues, comprehensive tourism revenues, and total retail sales of social consumer goods, also increased due to low carbon development. Therefore, low-carbon tourism built up an industry chain system,

with energy saving and consumption reduction helping tourists to have a better experience, which further enabled island tourism to occupy a larger tourism market.

The "response" also occupied a crucial position with a 0.2088 weighted value. It included the actions of people to prevent or mitigate negative impacts on the island environment. Furthermore, the "response" was divided into government planning and corporate practice. However, the weighted value of government planning was 0.1448, which was twice as high as that of corporate practice. This shows that the government plays a crucial role in low-carbon activities. This is especially true when the government establishes enough standard documents for low-carbon development in the tourism field, and this will increase the value of low-carbon tourism. Moreover, establishing low-carbon tourism demonstration areas, increasing low-carbon publicity, and increasing the number of public bus operations are all efficient methods to increase the value of low-carbon tourism. Additionally, clean energy and energy-efficient equipment usage by corporates also influenced the island tourism evaluation with weighted values of 0.0359 and 0.0281, respectively.

The next elements were "pressure" and "state". The "pressure" was the direct impact of tourism activities on the island's natural environment. It was the root cause of environmental problems at a weighting of 0.1917. The "pressure" statement for the Changdao area was calculated based on the scale of island tourism, transportation, accommodation, and tourism activities. These were the reasons for local environmental changes and problems. The "state" weighted value was 0.1699. This reflected the status of the carbon emissions from the tourism industry on Changdao Island. In contrast with the other indicators, all the indicators at the "state" level were negative. This is due to the carbon emissions' negative effect on the regional

low-carbon development levels. All the "state" indicators had large weighted values for both the entropy weight and AHP methods. This is because carbon emissions are a key criterion for the island tourism environment.

The weight of the "driving forces" was 0.1469. They were the original deep-seated factors that affected the island's sustainable development. They usually refer to demographic, social, and economic changes that directly or indirectly affect economic changes. In this study, economic, social, resource, and geographical elements were chosen to identify the strength of the "driving forces". The geographical location was measured by the number of days with suitable temperatures for tourism. The geographical location's weighted value was 0.0184, which was smaller than the other indicators that were all larger than 0.04 at the "driving forces" level. The economic weighted value was 0.0450, which shows that economics could be one of the most important "driving forces".

Based on Table 1, the Entropy Weight and AHP Methods yielded almost identical weighting results. However, the Entropy Weight and AHP Method Weights of the economic indicators at the driving level were different. The Entropy Weight Method yielded a higher weight value than that which was obtained using the AHP method. 43 The Entropy Weight Method obtains data that is based on the value of the data because the regional economic income data 37 is a reflection of the regional development and contributes to the sustainable development of the region. Conversely, the weight of the economic income at the "impact" level that was obtained using the entropy weighting method was lower than that for the AHP method. The regional impact of low-carbon development features prominently in the economic income component, and therefore it received a high score for the AHP Method. In addition, the top five indicators with the highest weights were the sewage

treatment rate at the impact level, the number of normative documents related to low carbon development and the rate of clean energy usage in enterprises at the response level, and the transportation carbon emissions and total carbon emissions at the state level. The remaining weighted indicators with high values were mainly from the State and Response levels. Thus, it can be generalized that the government and enterprises' environmental protection actions have substantial value and significance for low carbon evaluation.

The low-carbon island tourism development evaluation index system comprised the objective layer (O), the criterion layer (C), the element layer (E), and the indicator layer (I). In terms of the criterion layer, the "impact" (C4) contributed the most to the low-carbon tourism development of Changdao Island, followed by the response (C5), pressure (C2), state (C3), and driving forces (C1). In terms of the element layer, the weights of the elements in descending order were as follows: the ecological environment (E12), government planning (E13), economic income (E11), carbon emissions from sub-sectors (E9), and carbon emissions from tourism (E10), and location (E4). Hence, the impact of tourism in Changdao Island on the ecological environment, local economy, and resident income was notable. The government support, planning, and management of low-carbon tourism can play an important role in promoting low-carbon tourism, and reducing the carbon emissions of the three core carbon source departments is the key to low-carbon tourism development. Moreover, the regional economic growth level will also become an important factor that drives the choice of tourists regarding the tourism destination. Then, in terms of the indicators layer, the weights of the elements in descending order were as follows: the sewage treatment rate (I41), rate of clean energy usage (I48), number of normative documents related to low-carbon development (I44), carbon emissions from tourism (I30), and total carbon emissions from tourism

transportation (I27). These are in line with the main functions of environmental protection and the development direction of building an international zero-carbon demonstration zone in Changdao Island's comprehensive marine ecological civilization pilot zone.

5. Conclusion

This study selected Changdao Island as the focus of the study and based on the data from 2016 to 2021, the carbon emissions from tourism in Changdao Island were calculated and the evaluation results of the low-carbon tourism development level were analyed comprehensively. The main conclusions are specified below:

Low-carbon tourism is the basic requirement and correct direction for island tourism development. The development of tourism with a low carbon focus will build a protection network for the green and sustainable development of the island ecosystem and it will provide a driving force for the transformation of the island and fishing villages from a traditional development model to a modern development model. Moreover, it can revitalize local economic development and improve the incomes, living standards, and quality of life of the residents of the islands. Although the low-carbon tourism development level in Changdao Island is gradually increasing, there is still a large potential for improvement. The total carbon emissions from tourism in Changdao Island are decreasing, and the decrease is closely related to the reduction in the tourism market size. Hence, to promote island tourism and local economic growth and achieve the low-carbon development goal, attention should be paid to how to decouple the relationship between the tourism economy and carbon emissions.

In light of the three sub-sectors of tourism, the carbon emissions from tourism in Changdao

Island were mainly due to tourism transportation and tourist accommodation. When compared with the carbon emissions from other tourism destinations, the carbon emissions from tourism transportation in Changdao Island made up a lower proportion, which was mainly because tourists can only enter the island by ship, which is a central transportation mode with low unit carbon emissions. Additionally, since the end of 2018, private tourist cars were banned from entering Changdao Island, so tourists can only enter the island by ship and use electric cars, shared bicycles, electric bicycles, public electric buses, taxis, walking, and other available vehicles for internal transportation. This promotes carbon reduction. However, since the implementation of the vehicle policy in 2018, the tourism person-time decreased for the first time in 2019 after a continuous slow increase. The ban on private cars has had certain negative impacts on the number of tourists choosing Changdao Island as a tourism destination and constrains tourism development on Changdao Island. Hence, learning from the experience of mass tourism transport planning in coastal areas, island-hopping routes should be built based on factors such as attractions, tourism facilities, and geographical conditions, and convenient tourism services should be introduced, such as tourist sightseeing buses and self-drive electric car hire and charging services.

The carbon emissions from tourist accommodation made up a relatively higher proportion. The accommodations are limited by the high costs of clean energy, and fishtainment homestay operators have a low rate of clean energy usage. In light of this, relevant government departments should further encourage and support the use of clean energy in the tourism accommodation sector by increasing clean energy inputs and providing financial incentives while promoting the special fishtainent homestay construction project on Changdao Island. Furthermore, environmentally friendly advertisements, points rewards, or gifts can increase visitors’ awareness of conserving

resources and reducing the use of disposable items.

When compared with China's Hainan Island, South Korea's Jeju Island, Japan's Enoshima Island, and Indonesia's Bali Island, Changdao Island's tourism development had a single form, tourism consumption was weak, and it had a low impact on the local economy and residents' incomes.

Since this study involves county-level indicators and the type of indicators changes considerably every five years, there are limitations to the calculation of the carbon emissions from island tourism and the construction of evaluation indicators for low-carbon tourism. However, the findings of this study could be used to inform low-carbon development in Changdao Island and the evaluation system could be used as a basis for systems on other islands.

hhh

تقرير الاصالة

18%	6%	17%	%
مؤشر التشابه	مصادر الانترنت	الاصدارات	مستندات الطالب

المصادر الرئيسية

1	Shuai Mao, Haihong Li, Xiang Cai, Fengqin Liu, Hong Zhong, Yang Song. "Evaluation of the Development Level of Low-Carbon Tourism in Chinese Cities", Frontiers in Environmental Science, 2022<br>الاصدارات	1%
2	nagasaki-u.repo.nii.ac.jp<br>مصدر الانترنت	1%
3	link.springer.com<br>مصدر الانترنت	1%
4	Xiaojun Ma, Miaomiao Han, Jian Luo, Yanqi Song, Ruimin Chen, Xueying Sun. "The empirical decomposition and peak path of China’s tourism carbon emissions", Environmental Science and Pollution Research, 2021<br>الاصدارات	1%
5	LTLGB 2012, 2013.<br>الاصدارات	1%

6	"Research Progress and Current Situation on Tourism Carbon Emissions", Environment, Resource and Ecology Journal, 2023<br>الاصدارات	1 %
7	www.researchgate.net<br>مصدر الانترنت	1 %
8	Yuh-Jen Cho, Yu Wang, Lisa Li-I Hsu. "Constructing Taiwan's Low-Carbon Tourism Development Suitability Evaluation Indicators", Asia Pacific Journal of Tourism Research, 2015<br>الاصدارات	1 %
9	Xiaopeng Si, Zi Tang, Weili Wang, Yan Liang. "Evaluation and influencing factors of the tourism industry efficiency under carbon emission constraints in China", Environmental Monitoring and Assessment, 2023<br>الاصدارات	1 %
10	Shasha Yang, Zhicheng Duan, Xiaokun Jiang. "Spatial dynamics and influencing factors of carbon rebound effect in tourism transport: Evidence from the Yangtze-river delta urban agglomeration", Journal of Environmental Management, 2023<br>الاصدارات	<1 %
11	"High Performance Networking, Computing, and Communication Systems", Springer	<1 %

Science and Business Media LLC, 2011
الاصدارات

12 Tian Liu, Tianchao Yang. "Study on Carbon Emission and Its Influencing Factors in China’s Tourism Industry", Polish Journal of Environmental Studies, 2024
الاصدارات
<1 %

13 www.francis-press.com
مصدر الانترنت
<1 %

14 Ahmad Zuhairi Abdullah, Azlin Fazlina Osman. "Advances in Energy, Environment and Chemical Engineering", CRC Press, 2022
الاصدارات
<1 %

15 Bo Chen, Wenling Tang, Zhida Chen, Xiyuan Yang. "Spatiotemporal Variation in Carbon Emissions in China’s Tourism Industry during the COVID-19 Pandemic and Ecological Compensation Mechanism", Sustainability, 2023
الاصدارات
<1 %

16 mdpi-res.com
مصدر الانترنت
<1 %

17 www2.mdpi.com
مصدر الانترنت
<1 %

18 Yan Jinming, Xia Fangzhou. "Comprehensive Land Consolidation in China", Routledge, 2023
الاصدارات
<1 %

19	dokumen.pub<br>مصدر الانترنت	<1 %
20	Mingwei Li, Bingxue Shao, Xiasheng Shi. "Impact of High-Speed Rail on the Development Efficiency of Low-Carbon Tourism: A Case Study of an Agglomeration in China", Sustainability, 2022<br>الاصدارات	<1 %
21	Jianping Zha, Jiaquan Dai, Siqi Ma, Yirui Chen, Xiaohui Wang. "How to decouple tourism growth from carbon emissions? A case study of Chengdu, China", Tourism Management Perspectives, 2021<br>الاصدارات	<1 %
22	mdpi.com<br>مصدر الانترنت	<1 %
23	Lian Da, Yang ShiQiang, Yang Wu, Ran WenRui, Zhang Min. "Prediction of Carbon Emissions in Guizhou Province-Based on Different Neural Network Models", Cold Spring Harbor Laboratory, 2023<br>الاصدارات	<1 %
24	Young-Jin Cha. "Advances in Urban Construction and Management Engineering", CRC Press, 2023<br>الاصدارات	<1 %
25	www.hindawi.com	

مصدر الانترنت

<1%

26 Chao Chen, Weiping Wu. "Threshold Effects of Digital Economy on Tourism Carbon Emissions: Empirical Evidence from the Yangtze River Economic Belt in China", Polish Journal of Environmental Studies, 2024

الاصدارات

<1%

27 Proceedings of 20th International Conference on Industrial Engineering and Engineering Management, 2013.

الاصدارات

<1%

28 Rui Cao, Yanhua Mo, Jiangming Ma. "Carbon Footprint Analysis of Tourism Life Cycle: The Case of Guilin from 2011 to 2022", Sustainability, 2023

الاصدارات

<1%

29 Can Huang, Jin-Wei Wang, Chien-Ming Wang, Jin-Hua Cheng, Juan Dai. "Does tourism industry agglomeration reduce carbon emissions?", Environmental Science and Pollution Research, 2021

الاصدارات

<1%

30 Hongliang Li, Dong Li, Yunhua Li. "A multi-index assessment method for evaluating coverage effectiveness of remote sensing

<1%

satellite", Chinese Journal of Aeronautics, 2018
الاصدارات

31 Yiming Liu, Sunhee Suk. "Constructing an Evaluation Index System for China's Low-Carbon Tourism Region—An Example from the Daxinganling Region", Sustainability, 2021 <1%
الاصدارات

32 Pu Wu, Peihua Shi. "An estimation of energy consumption and CO2 emissions in tourism sector of China", Journal of Geographical Sciences, 2011 <1%
الاصدارات

33 Huiqiang Ma, Jiale Liu, Jianchao Xi. "Decoupling and decomposition analysis of carbon emissions in Beijing's tourism traffic", Environment, Development and Sustainability, 2021 <1%
الاصدارات

34 www.frontiersin.org <1%
مصدر الانترنت

35 Liguo Wang, Haoxiang Zhao, Jiawei Liu, Taiyi He, Hai Zhu, Yiming Liu. "How does the digital economy affect carbon emissions from tourism? Empirical evidence from China", Journal of Cleaner Production, 2024 <1%
الاصدارات

36	iieta.org<br>مصدر الانترنت	<1 %
37	www.rochford.gov.uk<br>مصدر الانترنت	<1 %
38	Chen Gong, Qingyue Tan, Guobin Liu, Mingxiang Xu. "Positive effects of mixed-species plantations on soil water storage across the Chinese Loess Plateau", Forest Ecology and Management, 2024<br>الاصدارات	<1 %
39	Chongfu Huang, Abdelouahid Lyhyaoui, Zhai Guofang, Nesrin Benhayoun. "Emerging Economies, Risk and Development, and Intelligent Technology - Proceedings of the 5th International Conference on Risk Analysis and Crisis Response, June 1-3, 2015, Tangier, Morocco", CRC Press, 2019<br>الاصدارات	<1 %
40	Jie Su. "Impact of tourism resource development based on low-carbon mode: a case study of Guizhou ethnic areas", Ecological Processes, 2019<br>الاصدارات	<1 %
41	Weijun Wang, Hongshan Zhang. "Evaluation of Low Carbon Economy Development in China's Provinces: Based on Entropy-OWA Algorithm and Grey Correlation Improvement	<1 %

TOPSIS", IOP Conference Series: Earth and Environmental Science, 2019
الاصدارات

42 Yuan Chi, Honghua Shi, Yuanyuan Wang, Zhen Guo, Enkang Wang. "Evaluation on island ecological vulnerability and its spatial heterogeneity", Marine Pollution Bulletin, 2017 <1%
الاصدارات

43 www.mdpi.com <1%
مصدر الانترنت

44 Chengcai Tang, Linsheng Zhong, Pin Ng. "Factors that Influence the Tourism Industry's Carbon Emissions: a Tourism Area Life Cycle Model Perspective", Energy Policy, 2017 <1%
الاصدارات

45 Li Youmei. "China’s Middle Class - The New Social Stratum", Routledge, 2021 <1%
الاصدارات

46 Li, Xiao Qin, Yuan Yin, and Hai Xia Lu. "The Building of Scenic Area Evaluation Index System on Low-Carbon Economy Perspective", Advanced Materials Research, 2012. <1%
الاصدارات

47 Meiwei Tang, Shouzhong Ge. "Accounting for carbon emissions associated with tourism- <1%

related consumption", Tourism Economics, 2018
الاصدارات

48 Tianjiao Li, Huimin Wang, Zhou Fang, Gang Liu, Fan Zhang, Haitao Zhang, Xuxia Li. "Integrating river health into the supply and demand management framework for river basin ecosystem services", Sustainable Production and Consumption, 2022
الاصدارات
<1 %

49 Zhang, Wei Hua, Jian Hua Zhang, and Rui Li. "Assessment about Development Level of Low-Carbon Economy in Resource-Based City", Applied Mechanics and Materials, 2014.
الاصدارات
<1 %

50 pubmed.ncbi.nlm.nih.gov
مصدر الانترنت
<1 %

51 "Use of Household Survey Data as a Tool to Assess the Carbon Footprint of Rural Tourist Accommodation and Related Services in China: A Case Study of Mount Qingcheng", Sustainability, 2017
الاصدارات
<1 %

52 Burak Hasirciolu, Jesus Gomez-Vilardebo, Deniz Gunduz. "Bivariate Polynomial Codes for Secure Distributed Matrix Multiplication", IEEE Journal on Selected Areas in Communications, 2022
<1 %

الاصدارات

53 Gang Liu, Pengfei Shi, Feng Hai, Yi Zhang, Xingming Li. "Study on Measurement of Green Productivity of Tourism in the Yangtze River Economic Zone, China", Sustainability, 2018 <1%
الاصدارات

54 Jianhua Zhu, Luxin Wan, Huijuan Zhao, Longzhen Yu, Siyu Xiao. "Evaluation of the integration of industrialization and information-based entropy AHP–cross-efficiency DEA model", Chinese Management Studies, 2023 <1%
الاصدارات

55 Junyi Zhang, Cheng-Min Feng. "Routledge Handbook of Transport in Asia", Routledge, 2018 <1%
الاصدارات

56 Li, Shi, Han, Deng, Liu. "Measurement and Spatial Variation of Green Total Factor Productivity of the Tourism Industry in China", International Journal of Environmental Research and Public Health, 2020 <1%
الاصدارات

57 Li, Jiong Hua, Rosalind Sia, and Li Yang. "Study on Low-Carbon Development in the Eco-Tourism Area: Taking Qinghai Lake of <1%

China as an Example", Advanced Materials Research, 2012.
الاصدارات

58 Mei, Yan, and Geng Yang. "Study on the Development of Low-Carbon Tourism", Advanced Materials Research, 2012. <1 %
الاصدارات

59 Tang, Chengcai, Linsheng Zhong, Wenjing Fan, and Shengkui Cheng. "Energy consumption and carbon emission for tourism transport in World Heritage Sites: a case of the Wulingyuan area in China : Energy consumption and carbon emission for tourism transport in World Heritage Sites: a case of the Wulingyuan area in China", Natural Resources Forum, 2015. <1 %
الاصدارات

60 Wei Zhang, Ying Zhan, Ruiyang Yin, Xunbo Yuan. "The Tourism Eco-Efficiency Measurement and Its Influencing Factors in the Yellow River Basin", Sustainability, 2022 <1 %
الاصدارات

61 Yao Liu, Jing Liu, Wenbei Yu, Yao Peng, Wei Yan, Yu Li, Jiujun Zhang. "Hollow spherical ZnO with mesoporous shell for highly enhanced gas sensitivity and selectivity", Chemistry – An Asian Journal, 2022 <1 %
الاصدارات

62 Zhang, Ying, and Qiao Zhou. "Research on Developing Strategy of the Ancient City Pingyao Based on Low-Carbon Tourism", Advanced Materials Research, 2012.
الاصدارات
<1 %

---

63 Zhongxu Zhao, Ying Pan, Jing Zhu, Junxi Wu, Ran Zhu. "The Impact of Urbanization on the Delivery of Public Service–Related SDGs in China", Sustainable Cities and Society, 2022
الاصدارات
<1 %

---

64 Zhongyu Bai, Qichuan Ding, Hongli Xu, Jianning Chi, Xiangyue Zhang, Tiansheng Sun. "Skeleton-based similar action recognition through integrating the salient image feature into a center-connected graph convolutional network", Neurocomputing, 2022
الاصدارات
<1 %

---

65 downloads.hindawi.com
مصدر الانترنت
<1 %

---

66 LISS 2014, 2015.
الاصدارات
<1 %

---

67 Weiqing Meng, Lingying Xu, Beibei Hu, Jun Zhou, Zhongliang Wang. "Quantifying direct and indirect carbon dioxide emissions of the Chinese tourism industry", Journal of Cleaner Production, 2016
الاصدارات
<1 %

استثناء الاقتباسات	تشغيل	استثناء التطابقات	< 7 words
استثناء المراجع	تشغيل		
